# Reproducible orchestration of best practices for reaction path optimization with the nudged elastic band^1^^✰✰^^★^^★★^^★★★^

**DOI:** 10.1016/j.mex.2026.103899

**Published:** 2026-04-03

**Authors:** Rohit Goswami

**Affiliations:** Institute IMX and Lab-COSMO, École polytechnique fédérale de Lausanne (EPFL), Station 12, CH-1015 Lausanne, Switzerland

**Keywords:** Reaction path optimization, Snakemake, Nudged elastic band, Reproducibility, Workflow automation, High performance computing

## Abstract

The nudged elastic band (NEB) method is the standard approach for finding minimum energy paths and transition states on potential energy surfaces. Practical NEB calculations require several pre-processing steps: endpoint minimization, structural alignment, and initial path generation. These steps are typically handled by ad-hoc scripts or manual intervention, introducing errors and hindering reproducibility. We present a fully automated, open-source Snakemake workflow for small gas phase molecules that couples modern machine learning potentials (PET-MAD) to the eOn saddle point search software. Each step of the calculation lifecycle is encoded as an explicit dependency graph, from model retrieval and endpoint preparation through path initialization and band optimization. The workflow resolves all software dependencies from conda-forge, ensuring identical execution across platforms. Validation on the HCN to HNC isomerization demonstrates that the automated pipeline recovers the known single-barrier energy profile and product energy without manual intervention.

## Graphical abstract

**Figure:**
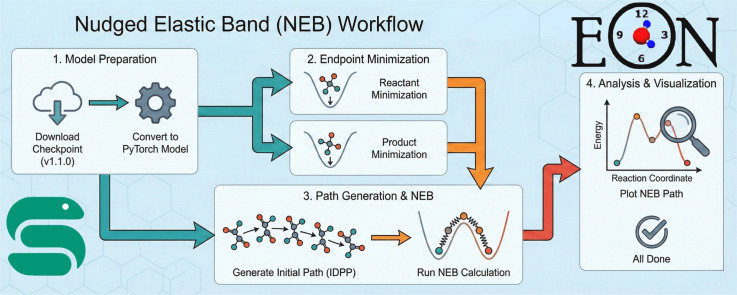

## Specifications table

**Table:** 

**Subject area**	ChemistryChemistry
**More specific subject area**	Reaction paths / Transition states / Computational chemistry
**Name of your method**	Nudged elastic band orchestrator
**Name and reference of original method**	[1] H. Jonsson, G. Mills, and K. W. Jacobsen, “Nudged elastic band method for finding minimum energy paths of transitions,” in Classical and Quantum Dynamics in Condensed Phase Simulations, World Scientific, 1998, pp. 385–404. doi: 10.1142/9789812839664_0016. [2] G. Henkelman, B. P. Uberuaga, and H. Jónsson, “A climbing image nudged elastic band method for finding saddle points and minimum energy paths,” J. Chem. Phys., vol. 113, no. 22, pp. 9901–9904, Nov. 2000, doi: 10.1063/1.1329672. [3] M. Gunde, N. Salles, A. Hémeryck, and L. Martin-Samos, “IRA: a shape matching approach for recognition and comparison of generic atomic patterns,” J. Chem. Inf. Model., vol. 61, no. 11, pp. 5446–5457, Nov. 2021, doi: 10.1021/acs.jcim.1c00567. [4] V. Ásgeirsson et al., “Nudged Elastic Band Method for Molecular Reactions Using Energy-Weighted Springs Combined with Eigenvector Following,” J. Chem. Theory Comput., vol. 17, no. 8, pp. 4929–4945, Aug. 2021, doi: 10.1021/acs.jctc.1c00462. [5] Rohit Goswami. Two-dimensional RMSD projections for reaction path visualization and validation. MethodsX, page 103,851, March 2026. ISSN 2215–0161. doi:10.1016/j.mex.2026.103851. [6] R. Goswami, “Efficient exploration of chemical kinetics,” Oct. 24, 2025, arXiv: arXiv:2510.21368. doi: 10.48550/arXiv.2510.21368. [7] F. Mölder et al., “Sustainable data analysis with Snakemake,” Apr. 19, 2021, F1000Research: 10:33. doi: 10.12688/f1000research.29032.2. [8] Rohit Goswami. Two-dimensional RMSD projections for reaction path visualization and validation. MethodsX, page 103,851, March 2026. ISSN 2215–0161. doi:10.1016/j.mex.2026.103851.
**Resource availability**	The workflow source code is available at https://github.com/HaoZeke/eon_orchestrator under the MIT license. A complete data archive containing all computed results, the ML potential model, per-iteration NEB data, and publication-quality figures is deposited on the Materials Cloud Archive. This resource is expected to facilitate rapid exploration of energy surfaces with scalable reproducible workflows. Part of the concepts described here are part of the atomistic cookbook as well.

## Introduction

The Nudged Elastic Band (NEB) method [1] finds the Minimum Energy Path (MEP) or the path of highest statistical weight in configuration space by optimizing a chain of N images connecting reactant and product endpoints. Each image experiences a projected potential force perpendicular to the path and a spring force parallel to it; the resulting “nudged” force prevents corner-cutting while maintaining image spacing. The climbing image modification [2] drives the highest-energy image to the first-order saddle point, and energy-weighted springs [3] concentrate images near the barrier region. Modern implementations combine band optimization with single-ended saddle search refinement for higher accuracy at the transition state [4,5] or even switch to locally approximated surfaces [[6], [7], [8]].

In practice, NEB calculations require careful preprocessing: endpoint structures must be minimized, atom orderings aligned, and an initial path generated that avoids atomic collisions. Mismatched atom labels or poor initial guesses cause outright failure or convergence to irrelevant saddle points. These steps are seldom integrated into NEB codes, leaving users to assemble ad-hoc scripts that are difficult to reproduce. We present an open-source Snakemake [9] workflow that automates this entire lifecycle, with all dependencies resolved from conda-forge via pixi. The workflow encodes each step as an explicit node in a dependency graph: endpoints are minimized and aligned, an initial path is generated via pairwise-potential interpolation, and only then does the NEB optimization begin. Users supply endpoint structures and a configuration file; the workflow handles the rest.

## The NEB orchestrator workflow

### Workflow as a directed acyclic graph

**Fig. 1:**
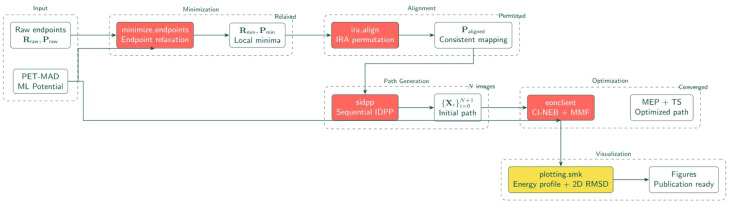
Workflow pipeline showing the Snakemake dependency graph. Raw endpoints undergo minimization and IRA alignment before SIDPP path generation. The hybrid CI-NEB-MMF optimization then refines the path to the MEP and transition state.

The workflow functions as a collection of interdependent rules defined in a **Snakefile**. The workflow is encoded as a directed acyclic graph (DAG) G=(V,E) where vertices represent rules (get_model, minimize, align, idpp, neb, visualize) and edges represent data dependencies (Fig. 1). Three properties follow directly:

### Automatic parallelization

Independent rules execute concurrently, e.g. minimize(R)∥minimize(P).

### Incremental recomputation

Modifying the reactant re-runs downstream rules but skips model retrieval and product minimization.

### Enforced ordering

neb←idpp←align←minimize←rawinputs.

Snakemake traverses this graph, executing rules only when dependencies are satisfied and inputs have changed.

## Automated dependency management

Beyond code, reproducibility requires pinning the workflow itself, including the models. The get_model.smk rule fetches a specific version of the PET-MAD [10] checkpoint from HuggingFace (lab-cosmo/upet), converts it to a deployable .pt file via mtt export (metatrain), and marks the output as protected against accidental deletion. Alternative models are supported through the same metatomic interface [11], requiring only a configuration change. Other models supported by eOn^1^ are also a parameter away.

## Endpoint preparation

Consistent atom-to-atom mapping between reactant and product is essential. The workflow implements a two-stage preparation process.

## Minimization

The minimize_endpoints rule relaxes each endpoint structure to the nearest local minimum on the chosen potential energy surface, preventing the NEB band from stalling on steep gradients at its ends. Configuration parameters:

### Optimizer

Limited memory Broyden–Fletcher–Goldfarb–Shanno [12].

### Force convergence

0.0514221 eV/Å∖ (10${}^{-3}$ Ha/Bohr).

### Maximum iterations

2000

### Maximum movement

0.1 Å

## Alignment

The workflow implements the Iterative Rotational Alignment (IRA) in two stages (Fig. 3):

**Fig. 2:**
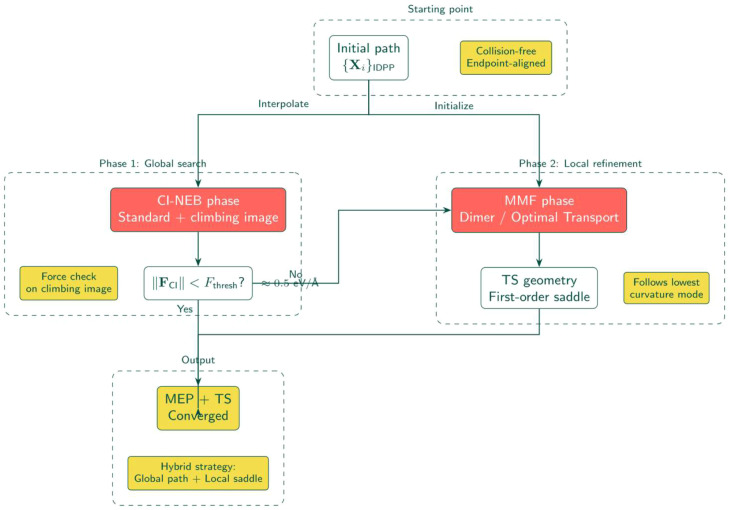
Hybrid CI-NEB-MMF optimization strategy. The CI-NEB phase performs global path optimization with a climbing image. When the climbing image force falls below threshold (≈0.5 eV/Å), the method switches to MMF for local saddle refinement via the lowest curvature mode.

**Fig. 3:**
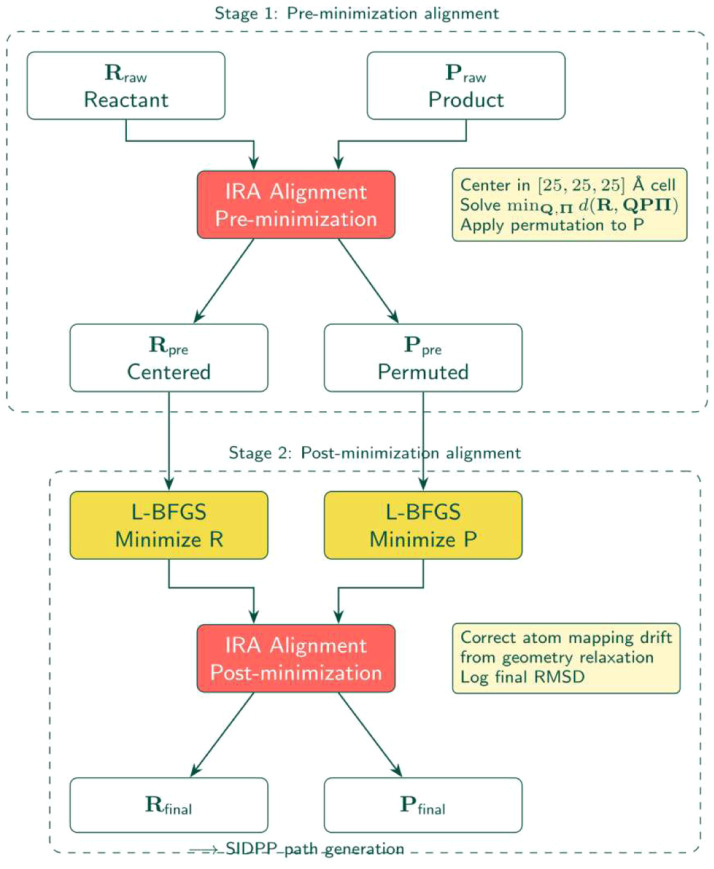
Two-stage IRA alignment process. Raw endpoints are centered and aligned before minimization to establish consistent atom ordering. After geometry relaxation, alignment is reapplied to correct any atom mapping drift introduced during optimization. Both stages solve the joint rotation-permutation problem via the IRA algorithm.

### Pre-minimization

Raw endpoints centered in [25, 25, 25] Å cell, aligned using IRA with k_max_=1.8 Å^-1^ (configurable). Ensures consistent atom ordering before geometry optimization.

### Post-minimization

After endpoint relaxation, reapply IRA alignment to correct atom mapping drift during minimization. Log the final RMSD (Root mean squared deviation) between reactant and product for verification.

The ira_align module employs the Iterative Rotations and Assignments [13] method, calculating optimal rotation and permutation to minimize Hausdorff distance. For configurations X with N atoms, the permutation-invariant RMSD is:

$$d\left(X,X_{\text{ref}}\right)=\underset{Q\in SO\left(3\right),\Pi \in S_{N}}{\text{min}}\sqrt{\frac{1}{N}{∥X-QX_{\text{ref}}\Pi ∥}_{F}^{2}}$$

where Q is the optimal rotation matrix and Π the permutation matrix. Automating this step ensures the path starts from the shortest (Euclidean) distance between endpoints in configuration space.

### Initial path generation

**Fig. 4:**
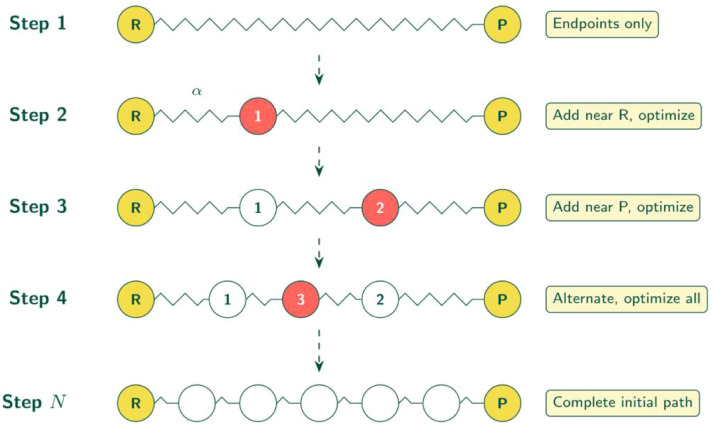
Sequential IDPP (SIDPP) path growth. Unlike standard IDPP which interpolates all images simultaneously, SIDPP adds one image at a time, alternating between reactant and product sides. After each addition, all intermediate images are re-optimized. The step size parameter α controls placement of each new image relative to the frontier. This sequential growth avoids local minima that trap simultaneous interpolation for complex reactions.

With aligned endpoints, the workflow generates an initial collision-free path using Sequential Image Dependent Pair Potential IDPP (SIDPP) [14]. Unlike standard IDPP [15] which interpolates all images at once then optimizes, SIDPP grows the path sequentially (Fig. 4):

1. Starting with only endpoints [R, P].
2. Add one image at a time (alternating reactant and product sides).
3. Optimize all intermediate images after each addition.
4. Repeat until target image count reached.

Sequential growth with intermediate optimization avoids local minima that trap standard IDPP for complex reactions. The step size parameter α=0.33 controls placement of each new image relative to the frontier. Standard IDPP is available as a fallback.

## Off-path CI-NEB (OCI-NEB) optimization

The optimization uses Climbing Image NEB (CI-NEB) [2] with energy-weighted springs [3] and minimum mode following (MMF) [[5], [7]] refinement as shown in Fig. 2. For image i with positions $R_{i}$, the NEB force is:

$$F_{i}^{\text{NEB}}=-\nabla E\left(R_{i}\right){\left|{}_{⊥}+F_{i}^{\text{spring}}\right|}_{∥}$$

where $\nabla E{|}_{⊥}$ is the potential force component perpendicular to the path tangent and $F^{\text{spring}}{|}_{∥}$ is the spring force projected along the tangent. The energy-weighted spring constant $k_{i}=k_{\text{min}}+\Delta k\text{max}\left(E_{i}-E_{\text{ref}},0\right)/\left(E_{\text{max}}-E_{\text{ref}}\right)$ concentrates images near the barrier by stiffening springs between high-energy images. The climbing image inverts the parallel force component at the highest-energy image $R_{\text{CI}}$:

$$F_{\text{CI}}=-\nabla E\left(R_{\text{CI}}\right)+2\left(\nabla E\left(R_{\text{CI}}\right)\cdot \hat{\tau }\right)\hat{\tau }$$

driving it uphill along the path tangent $\hat{\tau }$ while relaxing perpendicular to it. Once the climbing image converges, the workflow switches to off-path climbing image NEB (OCI-NEB) with MMF refinement along the lowest curvature mode.

Configuration parameters:

### Images

18 (including endpoints).

### Maximum iterations

1000

### Force convergence

0.0514221 eV/Å∖ (10${}^{-3}$ Ha/Bohr) [16].

### SIDPP growth (α)

0.33

### Climbing image activation

80% convergence.

### Energy-weighted springs

true (0.972–9.72 eV/Å${}^{2}$).

### Mode alignment with NEB tangent

0.8 (amount by which the rotated dimer can differ from).

All parameters are user-configurable, and Snakemake re-runs only those steps whose inputs have changed. The complete set of parameters are part of the accompanying repository and documented there.

## Applicability and parameter guidelines

The workflow is optimized for gas-phase molecular systems with 5–50 atoms. Table 1 provides default parameters and tuning guidelines for different system types.

**Table 1:** Suggested parameters.

Parameter	Small	Medium	Complex
	(5–20 atoms)	(20–50 atoms)	(50+ atoms)
NEB images	12–16	18–24	24–30
Force threshold (eV/Å)	0.051	0.051	0.026
IRA kmax (Å${}^{-1}$)	1.5–2.0	2.0–3.0	3.0–5.0
Cell size (Å)	20–25	25–30	30–40

Parameter selection principles:

### Number of images

Simple reactions (single bond break/form) need 12–16 images. Complex rearrangements with multiple bond changes or other intermediate minima may require 24–30 images for adequate path resolution.

### Force convergence

Standard threshold (0.051 eV/Å\ = 10^–3^ Ha/Bohr) suffices for most applications. High-precision kinetics studies should use 0.026 eV/Å\ (0.510^–3^ Ha/Bohr).

### IRA kmax

Controls permutation matching strictness. Well-behaved small molecules work with k_max_=1.5–2.0 Å^-1^. Difficult atom mapping (symmetric molecules, multiple identical fragments) may require k_max_=3.0–5.0 Å^-1^. The 2D RMSD visualization uses fixed kmax=14 Å^-1^ for consistency across systems. The IRA procedure becomes infeasible for extended systems in most cases without defining active regions.

### Cell size

Must be large enough to avoid periodic boundary artifacts. Default [25, 25, 25] Å works for most medium-sized molecules. Surface reactions or condensed-phase systems require modifications to handle periodic boundary conditions correctly, which is part of eOn natively, and so the workflow extends to these systems without modification.

## Method validation

To demonstrate that the workflow produces correct results from raw inputs without user intervention, we apply it to the HCN → HNC isomerization, a 3-atom proton transfer with a single transition state. CCSD(T)/cc-pCV5Z calculations [17] place the barrier at 2.09 eV and the HCN–HNC energy difference at 0.65 eV.

**Fig. 5:**
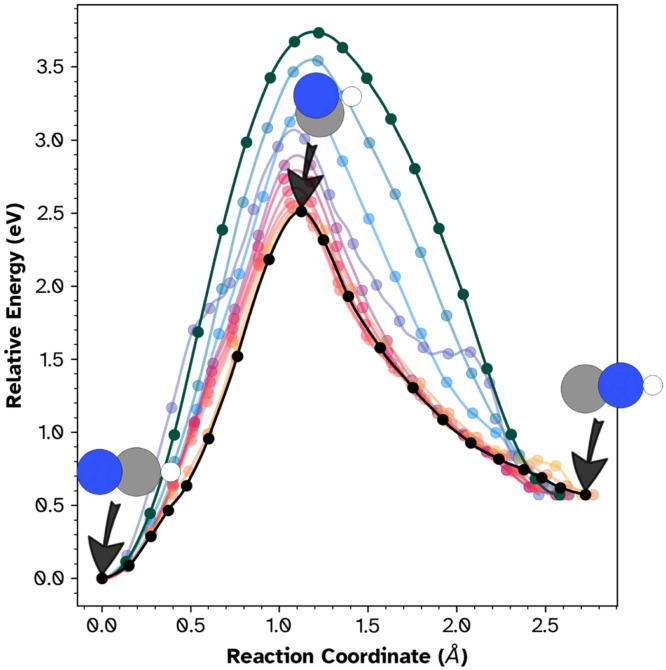
One-dimensional energy profile for the HCN → HNC isomerization. The single barrier of 2.46 eV separates the HCN reactant from the HNC product (0.57 eV above HCN). Colored traces show the optimization history from initial SIDPP guess (outer traces) to the converged path (black). Inset structures show the reactant, saddle point, and product geometries.

**Fig. 6:**
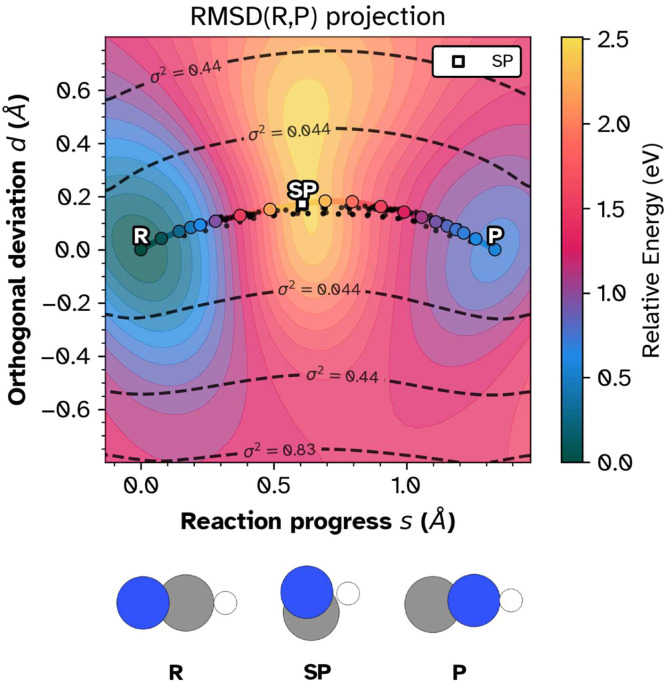
Two-dimensional RMSD landscape for the HCN → HNC isomerization. Axes represent permutation-invariant RMSD from reactant (reaction progress s) and orthogonal deviation (d). The color scale indicates interpolated energy. The reactant (R), saddle point (SP), and product (P) basins are clearly resolved.

Starting from endpoint structure files alone, the workflow completes minimization, IRA alignment, SIDPP path generation, and hybrid CI-NEB-MMF optimization. The resulting energy profile (Fig. 5) recovers the expected single-barrier topology with a barrier of 2.46 eV and a product energy 0.57 eV above the reactant. The 2D (s, d) [18] landscape (Fig. 6) resolves the reactant, saddle point, and product basins.

The repository includes four additional systems: the alanine dipeptide C7_eq_ → C5 conformational transition, Diels-Alder cycloaddition of ethylene and butadiene, the SN2 identity reaction F^-^ + CH_3F_, and vinyl alcohol to acetaldehyde tautomerization. Each requires only an endpoint pair and a YAML configuration file; the workflow handles alignment, path generation, and optimization identically. All five systems converge without manual intervention, and adding a new system amounts to providing two endpoint structures and editing a single configuration file.

## Reproduction

Complete reproduction proceeds as follows:



This generates plots and intermediates in the results folder. To reproduce the exact figures:



Documentation accompanies the repository with tutorial guidelines.

## Visualization framework

The workflow produces two complementary visualizations.

## One-dimensional energy profiles

Energy is plotted against a scalar reaction coordinate. Three variants are available:

### Image index

Simplest, plots $E_{i}$ vs. i∈[0,N]. Loses all geometric information about inter-image spacing.

### Cumulative path length

$s_{i}=\sum_{j=1}^{i}∥X_{j}-X_{j-1}{∥}_{2}$. Accounts for non-uniform image distribution but remains path-dependent.

### RMSD from reactant

$r_{i}=d\left(X_{i},X_{\text{react}}\right)$. Uses permutation-invariant RMSD; different methods can be compared on the same axis.

All profiles use the tangential forces $F_{∥,i}=-F_{i}\cdot {\hat{\tau }}_{i}$ to constrain the energy derivative during interpolation, producing physically consistent barriers .

## Two-dimensional RMSD landscapes

One-dimensional projections collapse the 3N-dimensional configuration space onto a single axis, obscuring path tortuosity and basin topology. The 2D framework projects onto intrinsic coordinates defined by the endpoints themselves .

Using the permutation-invariant RMSD from Eq. (1), we compute distances from each configuration X to both reactant (R) and product (P). The raw coordinates (r,p)=(d(X,R),d(X,P)) form a triangular region in the RMSD plane. A rigid rotation decomposes these into:

$\begin{array}{cc} s & =\text{reaction}\;\text{progress}\;(\text{along}\;\text{path})\\ d & =\text{orthogonal}\;\text{deviation}\;(\text{perpendicular}\;\text{to}\;\text{path}) \end{array}$

This decomposition separates forward progress from lateral excursions, analogous to path collective variables [19] but without smoothing parameters or training requirements.

The 2D landscape exposes path tortuosity, intermediate basins, and stagnation regions that 1D profiles collapse into featureless shoulders. The projection operates directly on Cartesian coordinates without training data; the $\approx 10^{3}$ geometries from a single NEB run suffice. Visualization code is provided in the chemparseplot and rgpycrumbs Python packages.

## Limitations

### ML potential accuracy

PET-MAD-XS provides point estimates without uncertainty quantification. Barrier heights should be validated with DFT single-point calculations for quantitative kinetics. Systems outside the training domain (transition metals, transition states [20], long range effect systems [21]) may require model finetuning.

### Permutation sensitivity

Two-stage IRA alignment ensures consistent endpoint mapping but does not guarantee correct permutations along the entire path. Reactions with many equivalent atoms or soft rotational modes warrant visual inspection of the initial path .

These limitations reflect the current state of ML potentials and optimal assignments of permutation-invariant alignments, not of the workflow itself.

## Conclusions

The workflow automates NEB calculations from raw endpoint structures through publication-quality visualizations, with every step declared as a node in a Snakemake DAG. The explicit dependency graph removes the manual setup that causes most NEB failures in practice: mismatched atom orderings, un-minimized endpoints, and poorly initialized paths. All dependencies resolve from conda-forge via pixi, so reproduction requires four commands. The workflow accepts any eOn compatible potential including the many metatomic interfaces and runs unchanged on a laptop or HPC cluster.

## Ethics statements

Not applicable.

## Related research article

N/A.

## Key innovations

• Automated Best Practices: Two-stage IRA alignment and SIDPP path generation enforce endpoint minimization and alignment before optimization begins.
• Dependency Management: Snakemake DAG automatically handles model versioning, retrieval, and incremental recomputation.
• HPC Scalability: Seamless scaling from local testing to cluster execution via Snakemake, with all dependencies resolved from conda-forge via pixi.

## CRediT authorship contribution statement

**Rohit Goswami:** Conceptualization, Methodology, Software, Visualization, Writing – original draft, Writing – review & editing.

## Declaration of competing interest

The authors declare that they have no known competing financial interests or personal relationships that could have appeared to influence the work reported in this paper.

## Data Availability

Github repository + Materials Cloud Archive.

## Footnotes

1 https://eondocs.org
